# Single Sublayer Reconstruction in Substrate-Supported WS_2_ Twisted Bilayers

**DOI:** 10.1021/acsnano.5c01308

**Published:** 2025-07-13

**Authors:** Hung-Chang Hsu, Yi-Han Lee, Hao-Yu Chen, Michael Schnedler, Ming-Yang Li, Rafal E. Dunin-Borkowski, Iuliana P. Radu, Philipp Ebert, Ya-Ping Chiu

**Affiliations:** 1 Department of Physics, 33561 National Taiwan University, Taipei 10617, Taiwan; 2 Graduate School of Advanced Technology, 33561 National Taiwan University, Taipei 10617, Taiwan; 3 Ernst Ruska-Centrum (ER-C-1), 28334 Forschungszentrum Jülich GmbH, Jülich 52425, Germany; 4 Taiwan Semiconductor Manufacturing Company, Hsinchu 30078, Taiwan; 5 Institute of Physics, Academia Sinica, Taipei 115201, Taiwan; 6 Institute of Atomic and Molecular Sciences, Academia Sinica, Taipei 10617, Taiwan

**Keywords:** van der Waals heterostructures, transition metal dichalcogenides, scanning tunneling microscopy, moiré superlattice, twisted bilayers

## Abstract

Marginally twisted WS_2_ bilayers undergo lattice reconstructions, but it is unclear if the distortion is equally distributed or confined to specific sublayers. Here, we use *in situ* combined noncontact atomic force microscopy with scanning tunneling spectroscopy to tune the probing depth to extract electronic and atomic lattice information for each sublayer separately. We find a lattice reconstruction unexpectedly confined to the WS_2_ layer in contact with graphite only, governed by transition metal dichalcogenide-substrate interactions, leading to a peculiar type of a ferroelectric domain wall.

## Graphical abstract

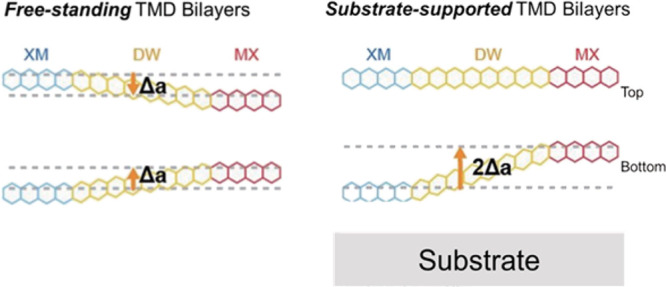

## Introduction

Van der Waals (vdW)-stacked two-dimensional multilayers are emerging as promising materials for the next generation ferroelectric memory and optoelectronic devices due to manifold opportunities to design and tune their electronic properties.^1–11^ Structural features, notably the local stacking configuration, are known to critically affect the electronic properties.^12–18^ Hence, a layer-resolved determination of lattice distortions is of utmost importance, but knowledge is scarce. Marginally twisted bilayer systems are known to undergo structural reconstructions,^12,19,20^ which tend to expand highly symmetric stacking configurations into commensurate domains at the expense of creating nanometer-confined strained domain walls (DWs), where the transition of the layer stacking takes place.^21–23^ This lattice reconstruction is identified to be equally distributed among both sublayers for free-standing bilayer WS_2_.^24^ However, no details are known for substrate-supported bilayer systems, despite the loss of symmetry that can be anticipated to induce a fundamentally different distribution of the lattice reconstruction among the different sublayers, thereby creating different types of electronic properties. The lack of layer-resolved probing of lattice relaxations is closely related to the complexity in accessing each sublayer separately with atomic resolution.

Here, we overcome this limitation by using *in situ* noncontact atomic force microscopy (nc-AFM) combined with scanning tunneling microscopy (STM) and spectroscopy (STS) applied to a twisted WS_2_ bilayer (tw-WS_2_ BL) on highly oriented pyrolytic graphite (HOPG). This combination allows us to tune the tip–sample separation rather freely, such that we extract atomic information from different depths below the topmost atomic layer. This provides a sublayer-resolved depth resolution enabling an identification of each sublayer’s atomic structure separately. We find a lattice reconstruction restricted surprisingly fully to the WS_2_ layer in contact with graphite only, while the top WS_2_ layer remains undistorted, resulting in a novel type of a ferroelectric Bloch domain wall with an additional Ising-like magnitude change. The results demonstrate the importance of the transition metal dichalcogenide–substrate interaction in the formation of asymmetric reconstructions, which opens the path to novel degrees of freedom and means for controlling electronic and ferroelectric properties in future devices.

## Results and Discussion

The WS_2_ film deposited on HOPG^25^ exhibits extended terraces, separated by steps (for preparation details see the Methods/Experimental Section). One example of a step is shown in the STM image in Figure 1A. The height profile across this step (inset) reveals a height difference of approximately 0.7 nm, consistent with the thickness of one WS_2_ monolayer (ML),^26^ but not compatible with an ML step of the HOPG substrate (0.35 nm). If the structure would arise from a bilayer step on the HOPG substrate, the electronic and structural properties of the WS_2_ film would be identical on both sides of the step. The scanning tunneling spectra in Figure 1B show that this is not the case. Thus, the step is attributed to an ML to bilayer (BL) transition in the WS_2_ film: On the BL WS_2_ (left terrace), the acquired tunneling spectrum, shown in light blue, reveals semiconducting properties with a band gap of about 2.4 ± 0.2 eV in line with the Γ-Q-band gap.^27,28^

**Figure 1:**
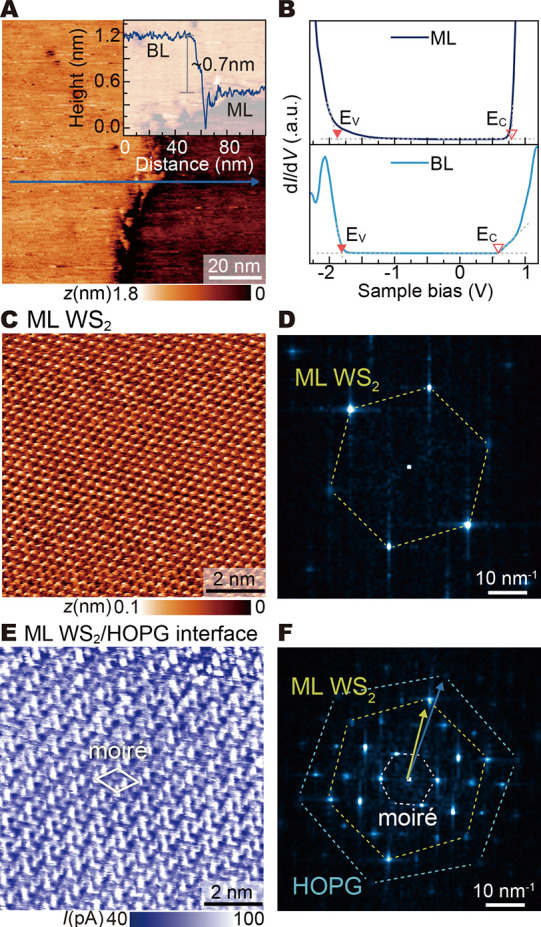
Sample overview and ML WS_2_–HOPG interface states. (A) STM image (sample bias +1 V, tunnel current 100 pA) showing a transition from (right) monolayer to (left) bilayer WS_2_/HOPG separated by a step height of ∼0.7 nm. (B) d*I*/d*V* tunneling spectra of ML and BL WS_2_. BL WS_2_ exhibits distinct states at the valence band edge and weaker at the conduction band edge, not present for ML WS_2_, which instead has a tail of states extending deep into the band gap. (C) Nc-AFM image (Δ*f* = +950 Hz) of the ML WS_2_ topography (background and drift corrected) and (D) corresponding FFT image, revealing only a WS_2_ honeycomb lattice. (E) Tunnel current image acquired at +0.4 V simultaneously with (C). (F) Corresponding FFT image, revealing a moiré pattern arising from a 4.6 ± 0.6° rotated stacking of ML WS_2_ on HOPG. The current image reveals gap states arising from a rehybridization of WS_2_ and HOPG states at the interface.

In addition, a distinct state appears close to the valence band edge (at about −2 V) and, albeit weaker, a hump close to the conduction band edge at about +0.7 V. The peak at −2 V has been previously attributed to the interaction of the two WS_2_ MLs forming the bilayer^29^ and is absent for the dark blue tunneling spectrum acquired on the ML WS_2_ (right terrace). The simultaneous appearance (for BL) and disappearance for ML of the weak hump at +0.7 V suggests that it may be of the same origin.

The dark blue spectrum of the ML WS_2_ exhibits an onset of filled (empty) states around −1.9 ± 0.4 V (+0.8 ± 0.1 V) and in between a band gap of about 2.7 ± 0.4 eV. The band gap is in good agreement with prior STS measurements of about 2.8 eV.^30,31^ For comparison with theory, we recall that electron tunneling occurs preferentially at the Γ point of the Brillouin zone, if DOS is available.^31^ For the conduction band, no DOS is available at the Γ point, but the valence band is only about 0.2–0.3 eV lower at the Γ point as compared to the K point (VBM).^32^ Hence, the 2.7 eV gap measured by STS corresponds to the (larger) Γ-K gap, yielding a fundamental band gap at the K point of about 2.4–2.5 eV, in good agreement with the calculated quasi particle band gap of 2.51 eV of a monolayer WS_2_.^32^

In addition, a tail of states extends ∼1 eV into the band gap at negative voltages. This tail can be attributed to the underlying states at the WS_2_ metallic-like HOPG interface. The interface states have a larger distance to the tip as compared with the WS_2_ layer itself. Due to the exponential tunnel current decay with separation, the tunnel current arising from interface states is smaller than that in the bands of the WS_2_ layer. Hence, at large sample voltages and thus large tip–sample separations, tunneling takes place primarily into/out of the conduction/valence band of WS_2_. However, when the tip is approached sufficiently close to the sample, tunneling into the localized interface states positioned below the WS_2_ layer is promoted. This can be achieved experimentally by reducing the set voltage to values within the fundamental band gap. Then, tunneling is only possible into states associated with the metallic-like HOPG substrate and the WS_2_–HOPG interface.^33,34^ Therefore, we utilized combined nc-AFM/STS to probe the atomic topography (Figure 1C) simultaneously with current imaging tunneling spectroscopy (CITS) maps (Figure 1E). Nc-AFM is sensitive to the atomic structure (i.e., topography) of the WS_2_ layer in the here used short-range repulsive force regime (Pauli repulsion), whereas STM/STS at voltages corresponding to energies deep in the band gap is sensitive to gap states associated with the WS_2_–HOPG interface. (Further discussion and supporting data can be found in Figures S1–S4)

The nc-AFM image in Figure 1C and its corresponding FFT image in Figure 1D reveal only a hexagonal atomic structure with a lattice constant of 0.32 nm, which is in excellent agreement with a WS_2_ (0001) 1 × 1 layer, with no moiré pattern. This suggests that the WS_2_ ML exhibits no structural distortions.

In contrast, the simultaneously acquired current image taken at a bias voltage of +0.4 V corresponding to tunneling into gap states only (Figure 1E) displays a complex pattern with a unit cell size about three times larger than that of the atomic structure. To understand the origin of this superstructure, we turn to the corresponding FFT image (Figure 1F), which reveals a series of consecutively stacked hexagons of spots. The three most relevant centered hexagons are marked by dashed lines. The largest hexagon (blue dashed line) has the smallest lattice constant of about 0.25 nm, corresponding to that of the HOPG (0001) (1 × 1) substrate. The next smaller hexagon (yellow dashed line) has a lattice constant in line with that of the WS_2_ (0001) 1 × 1 ML but rotated by 4.6 ± 0.6° relative to HOPG. The rotated stacking creates a moiré pattern, giving rise to the innermost hexagon of spots (white dashed line). This moiré pattern shows up only in electronic states associated with the WS_2_–HOPG interface but not in the atomic structure of the top WS_2_ ML. This suggests that the moiré pattern arises from a hybridization of WS_2_ and HOPG states at the interface.

The same methodology can be applied to elucidate the sublayer-resolved reconstruction of a twisted WS_2_ bilayer on the HOPG system. For this purpose, we focus on a region with a large moiré superstructure (Figure 2A), with a period length (λ_m_) of 13.5 nm (yellow dotted rhombus), which corresponds to a rotation angle of 1.4° between the two WS_2_ sublayers. Each unit cell contains two domains, appearing dark and bright in STM images and denoted MX and XM, respectively. The domains are framed by domain walls running along the connection lines between adjacent XX edge points of the moiré unit cell (yellow rhombus and dashed line). The assignment of the high-symmetry stacking configurations (i.e., MX, XM, and XX) is supported by the respective tunneling spectra. The d*I*/d*V* curves in Figure 2B reveal the largest band gap *E*_g_ of about 2.3 eV at the XX point, whereas the two domains exhibit band gaps of about 2.0–2.1 eV. The difference in *E*_g_ is mainly caused by a shift of the valence band edge (*E*_V_) (Figure 2B), in agreement with theory predicting a downward shift of the *E*_V_ at the Γ point of the Brillouin zone with increasing interlayer separations.^35–37^ The largest interlayer separation occurs for the stacking present at the XX point,^24,35^ where the interacting sulfur atoms of the two WS_2_ layers face each other directly. Thereby, the S–S interaction is the largest, widening the band gap. Within the domain areas, the S atoms are laterally displaced, and their interaction is reduced, reducing the interlayer spacing and band gap. The DW exhibits an interlayer spacing between that of the domain regions and the XX point,^24^ in line with the observation of a band gap slightly larger than that of the domains. These *E*_V_ and *E*_g_ fluctuations give rise to the moiré pattern visible in STM and d*I*/d*V* images (e.g., Figure 2A).

**Figure 2:**
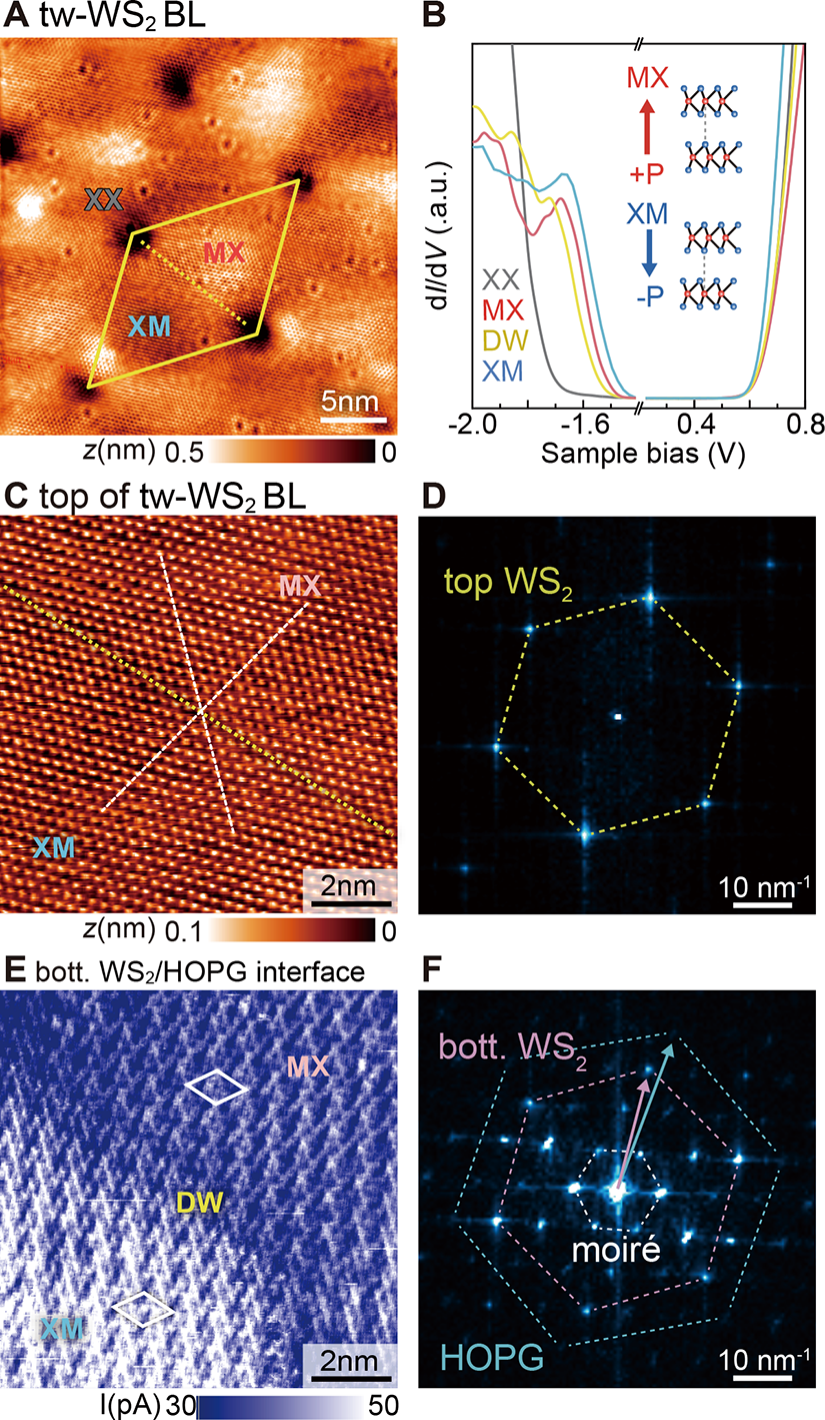
Distinction of top and bottom WS_2_ sublayers in tw-WS_2_ BL/HOPG. (A) STM image (−1.8 V, 1 nA) of a tw-WS_2_ BL/HOPG, showing a moiré superlattice with a period of 13.5 nm (yellow rhombus). (B) d*I*/d*V* tunneling spectra at different positions within the moiré pattern (with cropped band gap region, see Figure S10). The inset depicts the two stacking configurations of the MX and XM domains with opposite ferroelectric polarization. (C) Nc-AFM image (Δ*f* = +950 Hz) showing the atomic lattice of the top WS_2_ sublayer across an MX and XM domain border (background and drift corrected). The white dashed lines reveal that no lattice distortions occur. (D) Corresponding FFT image showing only the WS_2_ honeycomb lattice. (E) Simultaneously acquired tunneling current image probing gap states at the WS_2_–HOPG interface acquired at −0.9 V. (F) Corresponding FFT image reveals a moiré pattern (dashed white hexagon, real space unit cell marked by white rhombus in (e)) consistent with a 5.0 ± 0.4° twisted stacking of the bottom WS_2_ layer (pink hexagon) on HOPG (blue hexagon). In addition, higher-order spots occur. Note the striking similarity to the ML WS_2_–HOPG interface (Figure 1F).

In order to access separately the top and bottom WS_2_ layers, we turn to nc-AFM measurement in conjunction with STS. As outlined above, the atomically resolved nc-AFM image (Figure 2C) reveals the atomic lattice of the topmost WS_2_ layer, shown here for an area across an XM-MX domain wall. Visibly, the top WS_2_ layer of the tw-WS_2_ BL system exhibits no out-of-plane distortions within a detection limit of 0.02 nm. The dashed white lines marking atomic rows in Figure 2C reveal no in-plane lattice displacement between the two domains across the DW either. The corresponding FFT image (Figure 2D) also contains only spots related to a WS_2_ (0001) 1 × 1 atomic structure. Hence, the top WS_2_ layer of the tw-WS_2_ BL exhibits no structural distortions, like a free-standing WS_2_ layer.

However, a clear electronic moiré pattern is observable in STM/STS images, and a reconstruction is expected to occur for rotation angles below 3.5°.^37,38^ Therefore, all atomic displacements required for the reconstruction must occur in the bottom WS_2_ layer in contact with the HOPG substrate. For accessing the bottom WS_2_ layer, we reduce the tip–sample separation using voltages corresponding to energies within the band gap of the top WS_2_ layer. This allows tunneling into the gap states associated with the WS_2_–HOPG interface, in analogy to the case of one ML of WS_2_/HOPG in Figure 1E. Figure 2E illustrates the current image arising from tunneling out of all filled gap states with an energy ranging from the Fermi energy *E*_F_ to *E*_F_ = −0.9 eV. The gap states exhibit a different moiré pattern as compared to that of the top WS_2_ layer: The about 1 order of magnitude smaller moiré period length of 0.9 nm (moiré unit cell marked as white rhombus) corresponds to a rotation angle of 5.0 ± 0.4°. The corresponding FFT image in Figure 2F exhibits a striking similarity with that of the one ML WS_2_/HOPG in Figure 1F. One can recognize (i) an outermost hexagon of spots attributable to the HOPG substrate, (ii) a second hexagon of spots related to a lattice constant of WS_2_ rotated by 5.0 ± 0.4° with respect to the HOPG substrate, and (iii) finally, an inner hexagon of spots arising from the moiré pattern itself. Also note that the complex real space pattern in the current image of the gap states of the tw-WS_2_ BL/HOPG is similar to that of the ML WS_2_/HOPG (comparing Figure 2E with Figure 1E). This indicates that, in both cases, the hybridization of electronic states at the WS_2_–HOPG interface^26,34^ exhibits remarkable similarities, irrespective of the presence of a second WS_2_ layer stacked on top.

For a further analysis of the lateral in-plane atom displacements of the bottom WS_2_ sublayer, we enhanced the contrast of the image of the interface states in Figure 2E, by removing the current offset between both domains through Fourier filtering (Section S2). In order to understand the contrast of the resulting image (Figure 3A), we recall that, in the corresponding FFT image in Figure 2F, the spots of the WS_2_ (1 × 1) bottom sublayer and those of the moiré pattern exhibit the highest intensity. Hence, one can interpret the small protrusions in the current image as WS_2_ lattice, modulated by the moiré pattern.

**Figure 3:**
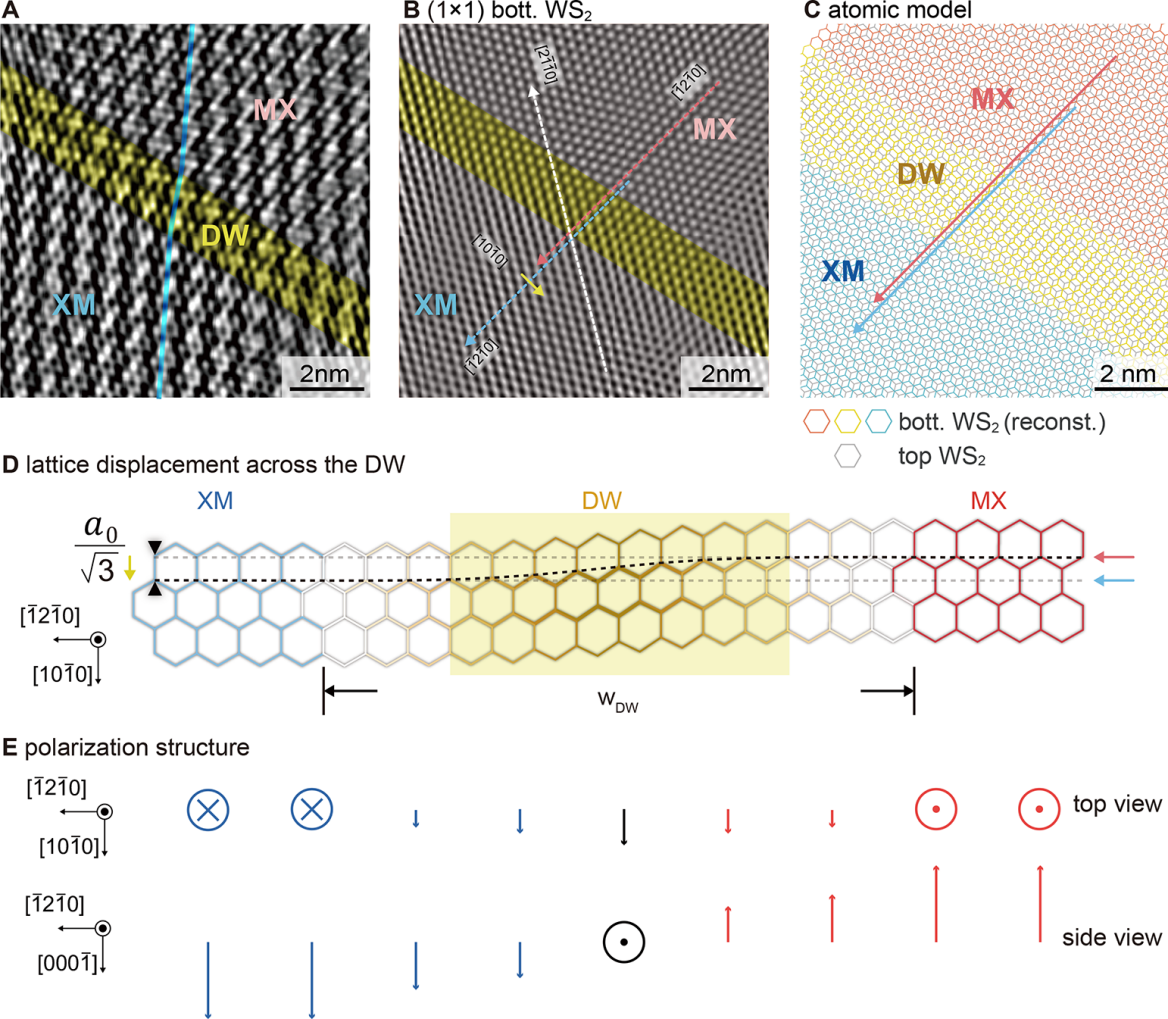
Lattice reconstruction of the bottom WS_2_ sublayer. (A) Current image obtained from Figure 2E after removal of the domain background offset using Fourier filtering, showing a displacement of the moiré pattern across the DW (blue line). (B) WS_2_ honeycomb lattice of the bottom WS_2_ layer obtained by Fourier filtering of Figure 2E, revealing a [101̅0] lattice shift of $\frac{a_{0}}{\sqrt{3}}$ , consistent with the shear strain displacement model across the DW illustrated in (D). (C) Model of the atomic lattice stacking of an undistorted top WS_2_ sublayer (gray honeycomb lattice) and a reconstructed bottom WS_2_ sublayer (blue, yellow, and red colored regions corresponding to the XM domain, DW, and MX domain, respectively). The lines indicate the lattice shift in accordance with that deduced from the current image in (B). (E) Schematic of the mixed Bloch-Ising polarization structure at the DW.

First, we focus on the moiré unit cells: The vertical blue line illustrates that the moiré unit cells are displaced in the MX domain relative to those in the XM domain. The displacement occurs in a narrow transition region limited to two full moiré unit cells (colored yellow). The presence of such a narrow DW of about 1.7 nm in comparison to domain sizes of 13.5 nm indicates that the XM and MX domains expanded at the expense of the DW. This can be rationalized in terms of total energy lowering through increasing the area of commensurate domains at the expense of concentrating the strain energy in a narrow highly strained DW area. Hence, the bottom WS_2_ sublayer exhibits a lateral distortion and reconstruction.

Second, we extracted the lattice of the bottom WS_2_ sublayer by Fourier transform filtering (Figure 3B). The dashed red and blue lines reveal that both domains are shifted by half a row separation in the [101̅0] direction, i.e., $\frac{a_{0}}{\sqrt{3}}$ , with *a*_0_ being the in-plane lattice constant. No displacement is detected in the [21̅1̅0] direction. Such lattice shifts occur across all 120° rotated DWs (Figure S6). Figure 3D schematically illustrates the observed shear distortion of the bottom WS_2_ sublayer. The lattice hexagons are colored in blue, yellow, and red to indicate the XM domain, DW, and MX domain regions, respectively. On this basis, we illustrate the atomic structure of the tw-WS_2_ BL system in Figure 3C. The undistorted atomic lattice of the top WS_2_ sublayer is shown as a perfect undistorted gray honeycomb lattice (as confirmed by the nc-AFM measurements in Figure 2C). This lattice is overlaid by the shear distorted bottom WS_2_ sublayer lattice, colored in blue, yellow, and red as in Figure 3D. The relative alignment is done by assuming S–W and W–S (hollow-type) stacking configurations in the XM and MX domains, respectively. The model based on an undistorted top and a reconstructed bottom WS_2_ sublayer exhibits a good agreement with the observed lattices.

This can be further corroborated by turning to the distortion of the moiré pattern at the DW. The experimental moiré pattern arising from the bottom WS_2_ layer and the HOPG substrate is extracted from Figure 2E using Fourier transform filtering and is displayed in Figure 4B. This is compared to a modeling based on the derived reconstruction of the bottom WS_2_ lattice and the undistorted HOPG with a rotation angle of 5.0° (Figure 4A) (see Section S4 and Figure S8). The model and experimental moiré patterns agree perfectly, quantifiable by three critical characteristics: (i) In the commensurate XM and MX domains, the moiré pattern has a regular hexagonal shape without distortion (see blue and red hexagons). (ii) The moiré pattern exhibits a lateral shift, Δ_shift_ = 0.28 nm, between the XM and MX domain (see red and blue dashed lines) in the experimental data as well as model. (iii) The moiré pattern is distorted in the DW region (see yellow triangle deviating from an equilateral triangle within the hexagons in the domains) (see Figure S7 revealing analogous shifts and distortions across every 120° rotated DWs).

**Figure 4:**
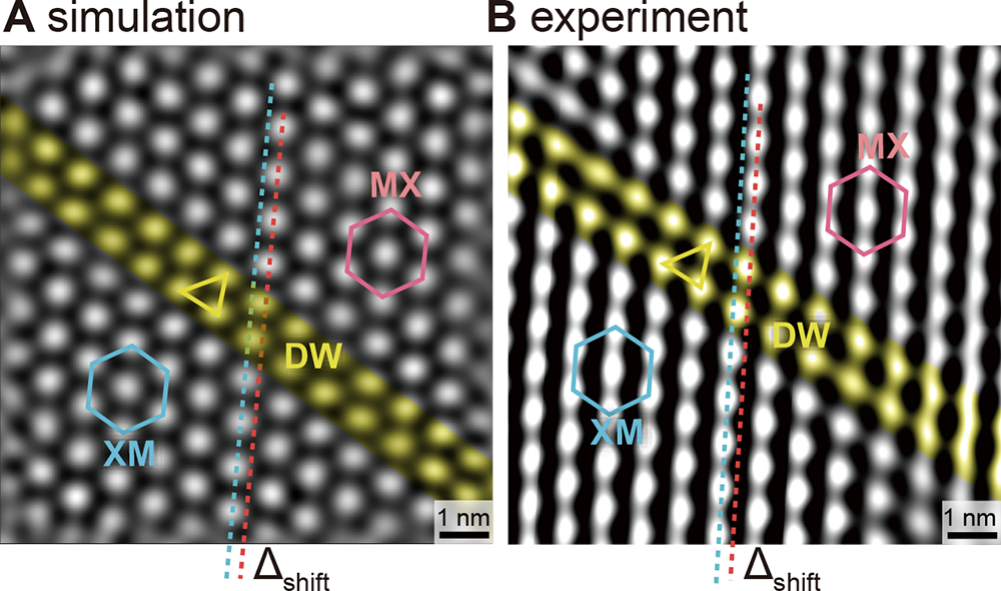
Effect of bottom sublayer reconstruction on the moiré pattern of WS_2_–HOPG interface states. Comparison of (A) a simulated moiré pattern across the DW and (B) an experimental one extracted through Fourier transform filtering from Figure 3A. The simulation is based on the model of the reconstructed bottom WS_2_ sublayer on HOPG shown in Figure 3C.

This agreement is only obtained for a structure, where all reconstruction induced distortions occur solely in the bottom WS_2_ layer, sandwiched between the ideal undistorted top WS_2_ sublayer and HOPG substrate. If one assumes that the atomic displacements induced by the reconstruction are equally distributed among both WS_2_ sublayers, no quantitative agreement is achieved (Figure S9), corroborating our finding of a reconstruction confined to only one WS_2_ sublayer.

The particular reconstruction of the bottom WS_2_ layer leads to a notable polarization structure across the DW schematically illustrated in Figure 3e. At the center of the DW, no out-of-plane polarization persists, but the lattice displacement results in an in-plane polarization oriented in the [101̅0] direction, i.e., along the domain wall. Thus, the ferroelectric domain wall exhibits a Bloch-type rotation of the polarization but combined with a change of the polarization magnitude. The change of magnitude differs, however, from that of the Ising DW since it does not become zero at the DW.

## Conclusions

In summary, we demonstrated a sublayer-resolved identification and quantification of an unexpected highly asymmetric reconstruction of marginally twisted bilayer WS_2_ on graphite. The in-plane lattice distortion is confined solely within the WS_2_ sublayer in contact with the graphite substrate and attributed to the transition metal dichalcogenide–substrate interaction. In contrast, the top WS_2_ sublayer remains unreconstructed, almost behaving like an ideal free-floating monolayer. The highly asymmetric reconstruction results in intriguing DW structures and ferroelectric properties, ultimately enabling a coupling with external electric fields and thus ferroelectric dynamics.

## Methods/Experimental

### Sample Preparation

For the fabrication of a twisted WS_2_ bilayer on highly oriented pyrolytic graphite, a 50 nm Bi metal layer was first deposited onto a CVD-grown WS_2_ monolayer (ML).^40^ A layer of PMMA (950k A2) was then spin-coated on top, and thermal release tape (TRT) was attached to serve as a pick-up stamp. The WS_2_ monolayer was mechanically lifted using the TRT/PMMA stack and then aligned and transferred onto a second CVD-grown WS_2_ monolayer with a controlled twist angle to form the twisted WS_2_ bilayer. This bilayer stack was subsequently transferred to a clean HOPG substrate. The TRT was released by heating on a 120 °C hot plate, and the PMMA layer was removed using acetone. Finally, the Bi metal was etched away with HNO_3_, followed by a rinse with isopropanol (IPA).^25^

### Nc-AFM, STM, and STS Measurements

After introducing the prepared sample into the ultrahigh vacuum chamber (pressure <10^–8^ Pa), high-resolution scanning probe microscopy measurements were performed at 77 K. All *in situ* measurements, including nc-AFM, STM, and STS, were conducted using a combined STM/QPlus sensor setup. STM imaging and STS differential conductance (d*I*/d*V*) spectra were acquired in the constant-current mode. In contrast, nc-AFM and tunneling current images were recorded in constant-frequency mode (sensor resonance frequency, *f*_0_ = 24 kHz; quality factor, *Q* = 6000), with the bias voltage applied to the sample. The AFM measurements were carried out in the repulsive short-range force regime, where the contrast is dominated by Pauli repulsion. Specifically, imaging was done at a frequency shift of Δ*f* = *f* – *f*_0_ = +950 Hz and +1100 Hz. In this regime, the AFM images are sensitive to atomic positions but not to local electrostatic potentials.

## Supplementary Material



## Notes

Separation of WS_2_ sublayers by bias-dependent imaging; illustration of the Fourier filter masks and processing steps; large scale nc-AFM and current images; modeling of moiré pattern of the bottom WS_2_/HOPG for various lattice alignments; full range of the tunneling spectra of tw-WS_2_ BL in Figure 2B (PDF)
