# Supplementary material for: Single Sublayer Reconstruction in Substrate-Supported WS2 Twisted Bilayers
Source: ACS Nano. 2025 Jul 13;19(28):25662–8. doi: 10.1021/acsnano.5c01308 (PMC12291578; doi:10.1021/acsnano.5c01308)
Supplement: Supplementary file 1 [file nn5c01308_si_001.pdf]

Supporting Information

Single sublayer reconstruction in substrate-supported WS_2 twisted bilayers

Hung-Chang Hsu[1], Yi-Han Lee[1], Hao-Yu Chen[2], Michael Schnedler[3], Ming-Yang Li[4], Rafal E. Dunin-Borkowski[3], Iuliana P. Radu[4], Philipp Ebert[3], Ya-Ping Chiu[1,2,5,6]

[1]Department of Physics, National Taiwan University, Taipei 10617, Taiwan
[2] Graduate School of Advanced Technology, National Taiwan University, Taipei 10617, Taiwan
[3]Ernst Ruska-Centrum (ER-C-1), Forschungszentrum Jülich GmbH, 52425 Jülich, Germany
[4]Taiwan Semiconductor Manufacturing Company, Hsinchu 30078, Taiwan
[5]Institute of Physics, Academia Sinica, Taipei 115201, Taiwan
[6]Institute of Atomic and Molecular Sciences, Academia Sinica, Taipei 10617, Taiwan

1. Separation of WS_2 sublayers by bias-dependent imaging

Fig. S1 illustrates for the one ML-WS_2/HOPG system that at large magnitudes of tunneling voltages (tunneling into valence band states, -1.9 V) current maps (**Fig. S1C**) exhibit the same WS_2(0001) 1×1 atomic structure as that visible in the respective nc-AFM image (**Fig. S1A**). This is also supported by the respective FFT pattern (compare **Fig. S1D and S1B**), whose most intense spots are due to the WS_2(0001) 1×1 atomic structure. All other spots related to WS_2-HOPG interface states are comparably weak.

In contrast, when the bias voltage is reduced to values corresponding to energies within the band gap (+0.4 V, tunneling into gap states only) a prominent moiré pattern appears **(Fig. S1E)**, which arises from the WS_2-HOPG interface states in the band gap. The respective FFT pattern in **Fig. S1F** highlights the strong appearance of moiré spots arising from the WS_2/HOPG interface.

The transition is further illustrated by line profiles through a 1×1 and a moiré spot in FFT patterns (**Fig. S2**). When the absolute tunneling voltage is reduced from large values (where tunneling into the bands dominates) to small values for in-gap tunneling only, the spots intensity changes from high intensity on 1×1 to high intensity of moiré spots.

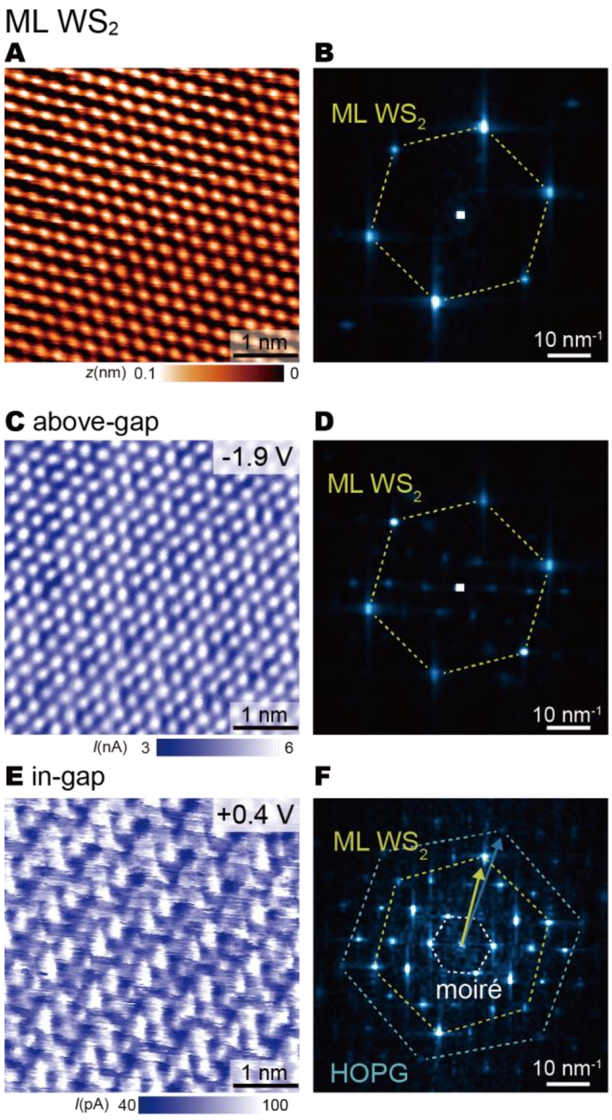

Fig. S1: Bias-dependent imaging of top layer topography vs. interface states of one ML WS_2/HOPG: (**A**) nc-AFM image (Δf=1100 Hz) and (**B**) corresponding FFT pattern, revealing the WS_2(0001) ML 1×1 atomic topography. (**C**) Tunneling current image acquired simultaneously at -1.9 V for tunneling out of valence band states and (**D**) corresponding FFT image, revealing a WS_2(0001) 1×1 atomic structure. (**E**) and (**F**) display the current image and corresponding FFT pattern for tunneling into the gap states at +0.4 V. These states reveal a pronounced moiré pattern arising from the twisted arrangement of the ML WS_2 and the HOPG substrate and interface states.

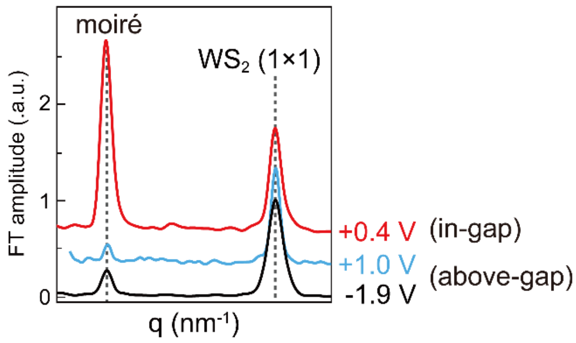

Fig. S2: Bias-dependent line profiles of FFT intensity across WS_2 (0001) 1×1 and moiré spots (normalized using the 1×1 spots' amplitude), supporting a switch in imaging the top layer topography at large voltages toward the interface states for in-gap tunneling at small voltages.

An analogous result is obtained for the twisted bilayer WS_2 on HOPG in **Figs. S3** and **S4**.

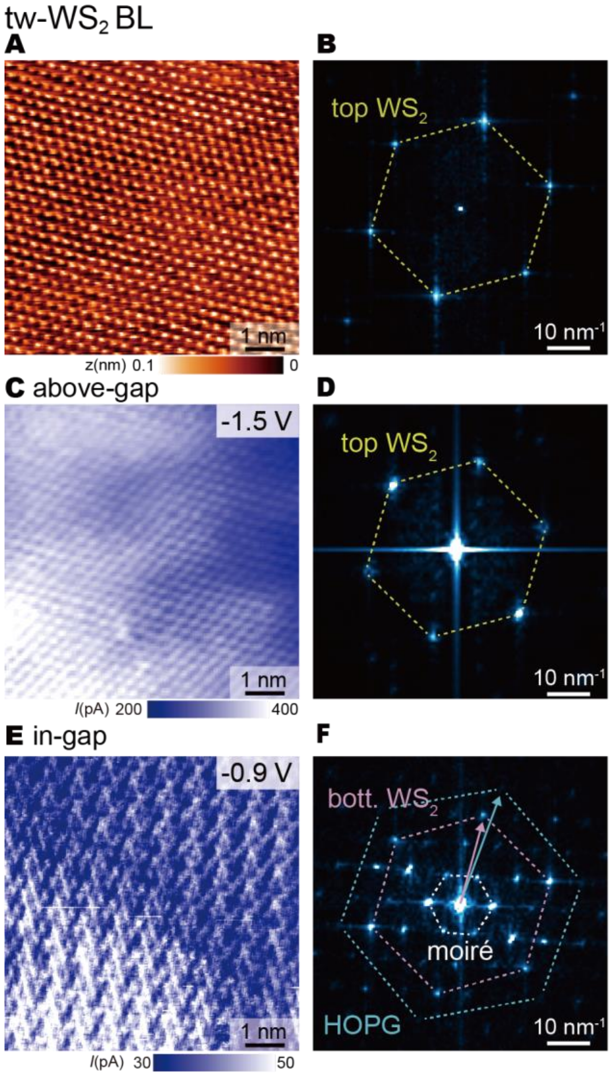

Fig. S3: Same as **Fig. S1**, but for the twisted-WS_2 bilayer (BL). (**A**) nc-AFM image (Δf=950 Hz) and (**B**) corresponding FFT pattern, revealing the WS_2(0001) ML 1×1 atomic topography. For large tunneling voltages the top layers 1×1 structure is revealed again **(C, D)**, whereas for in gap tunneling voltages the interface states between the bottom WS_2 layer and the HOPG substrate are probed **(E, F)**.

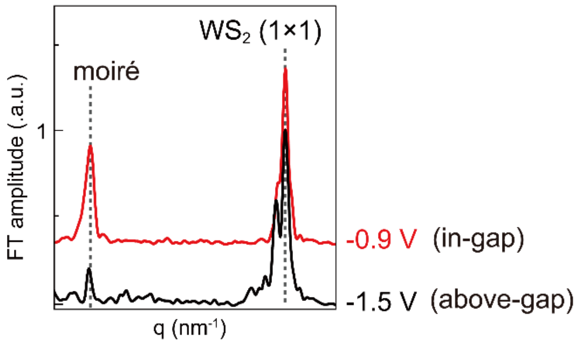

Fig. S4: Same as **Fig. S2** but for twisted BL WS_2.

2. Illustration of the Fourier filter masks and processing steps

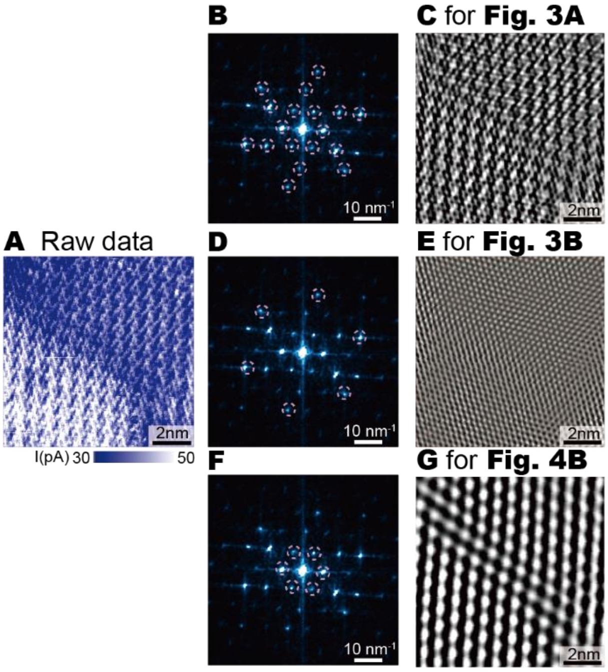

Fig. S5: Illustration of the Fourier filter masks (dashed circles) used in the Fourier filtering to obtain **Figs. 3A and B** and **Fig. 4B** in manuscript**.** The spots marked by circles were included in the inverse Fourier transform (circles represent the filter masks).

3. Large scale nc-AFM and current images

Fig. S6 demonstrates that the top WS_2 sublayer exhibits no lattice distortion, whereas in the bottom WS_2 layer the same atomic lattice shifts occur across all three 120° rotated domains in <10-10> directions. This result is in line with the example shown in **Fig. 3** of the manuscript and further supports that the lattice reconstruction is confined to the bottom WS_2 sublayer only.

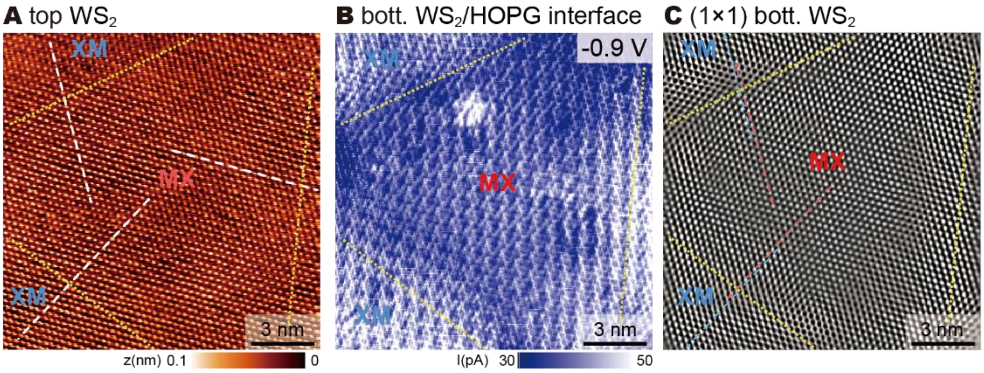

Fig. S6: Reconstruction of bilayer WS_2 on HOPG: (**A**) nc-AFM image of top WS_2 sublayer, (**B**) simultaneously acquired tunnel current image at -0.9 V revealing the in-gap states associated with the bottom WS_2/HOPG interface, and (**C**) honeycomb lattice of bottom WS_2 obtained by Fourier filtering from the current image (mask on the WS_2 1×1 spots). The data reveals no lattice distortion in the top WS_2 layer imaged by nc-AFM, whereas the bottom WS_2 sublayer exhibits lattice distortions across the domain walls. The white dashed lines in (**A**) reveal that no lattice distortions occur in the top WS_2 sublayer. The blue and red lines in (**C**) indicate the lattice displacement of the bottom WS_2 sublayer across the DWs, whose positions are indicated by the yellow dashed lines.

Fig. S7 illustrates the shift in the moiré pattern of the bottom WS_2/HOPG interface. Again this figure supports the results of **Fig. 4** of the manuscript, now for all three 120° rotated domains.

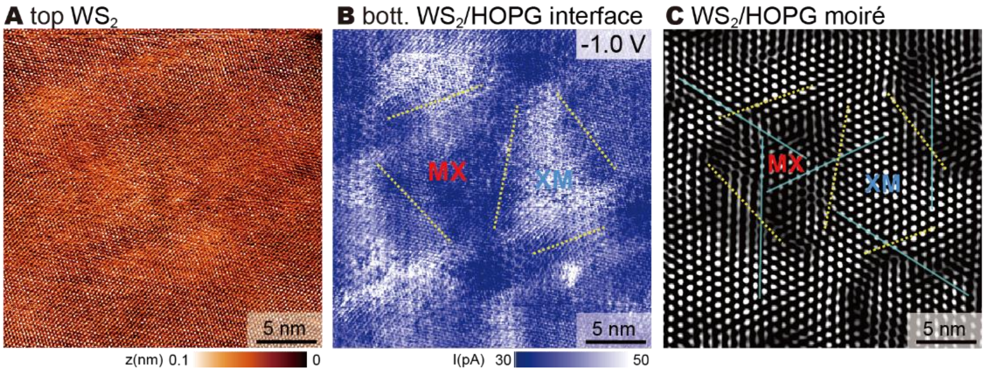

Fig. S7: (**A**, **B**) Same as **Fig. S6**, but with larger scanning range. (**C**) moiré pattern distribution extracted from the current image in (**B**) by Fourier filtering (mask on the moiré spots). The data reveals the same shifts of the moiré pattern across all domain wall as presented in **Fig. 4**, supporting the derived DW structure. The blue lines in (**C**) indicate the displacement of the moiré pattern across the DWs, whose positions are again indicated by the yellow dashed lines.

4. Modelling of moiré pattern of the bottom WS_2/HOPG for various lattice alignments

The moiré pattern is modelled by using two 2D Boolean arrays (shadow masks), which describe the atomic lattice positions (Boolean value = true) of the two twisted layers, respectively. The 2D Boolean arrays are then logically connected using an "AND" operator to obtain the moiré pattern. Further FFT filtering of the moiré period is used to reveal only the moiré superstructure. The process is visually shown in **Fig. S8**.

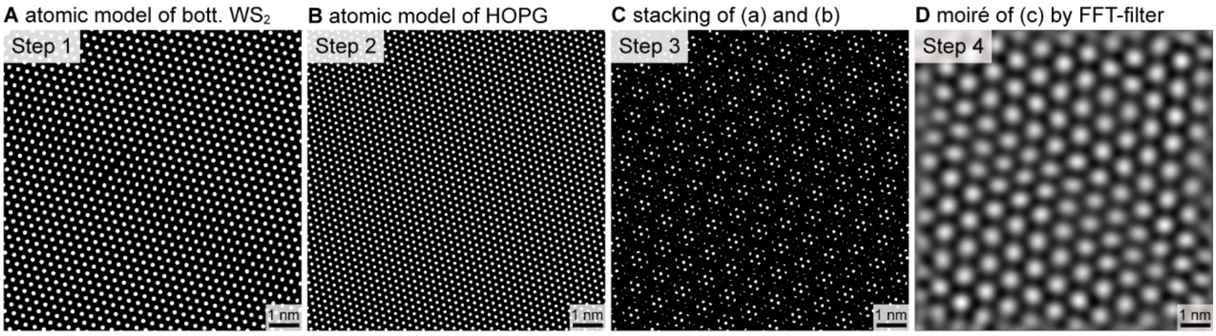

Fig. S8. Modelling of moiré superstructure for an area containing a DW: **(A)** Boolean array representing the atomic model of the bottom WS_2 layer corresponding to **Fig. 3(C)**. **(B)** Boolean array representing the atomic model of the HOPG substrate with lattice orientation determined by **Fig. 2(F)**. **(C)** AND operator applied to (A) and (B). (D) FFT-filtered image revealing the WS_2/HOPG moiré superstructure only.

On this basis the moiré pattern for different lattice alignments is simulated (Fig. S9).

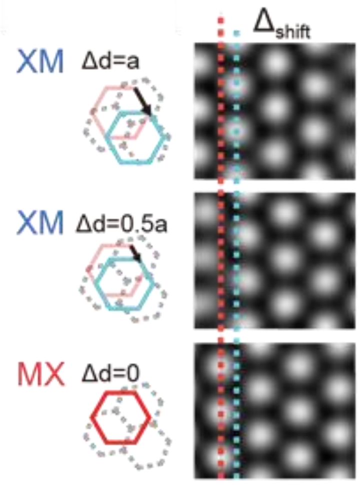

Fig. S9: Simulation of the moiré pattern for different lattice alignments of the bottom WS_2 sublayer and HOPG substrate. The moiré pattern exhibits a shift for different alignments illustrated by the dotted vertical lines.

5. Full range of the tunneling spectra of tw-WS_2 BL in Fig. 2B

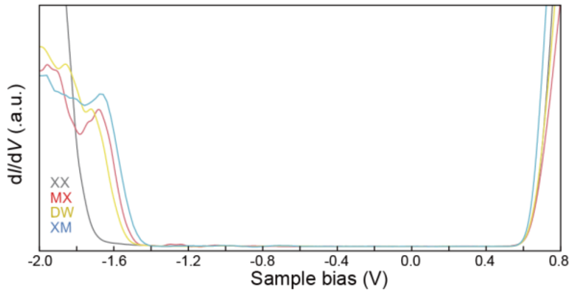

Fig. S10: STS curves of **Fig. 2B** without cropping data in x-axis.
